# Overexpressing Histone Deacetylase 5 in Rat Dorsal Striatum Alters Reward-Guided Decision-Making and Associated Neural Encoding

**DOI:** 10.1523/JNEUROSCI.0916-21.2021

**Published:** 2021-12-08

**Authors:** Heather J. Pribut, Daniela Vázquez, Alice D. Wei, Stephen S. Tennyson, Ian R. Davis, Matthew R. Roesch, Xuan Li

**Affiliations:** ^1^Department of Psychology, University of Maryland, College Park, Maryland 20742; ^2^ Neuroscience and Cognitive Science Program, University of Maryland, College Park, Maryland 20742

**Keywords:** decision, dorsal striatum, epigenetic, histone deacetylase 5, recording, reward

## Abstract

Accumulating evidence in the past decade implicates histone-modifying enzymes, such as class I histone deacetylases (HDACs), in learning and memory and, recently, habit formation. However, it is unclear whether HDACs play roles in complex cognitive function. To address this issue, we examined the role of dorsal striatal HDAC5, a class II HDAC, in reward-guided decision-making and associated neural encoding in rats. We first injected adeno-associated virus to overexpress a nuclear-localized HDAC5 in dorsal striatum (DS). We then recorded neural correlates from dorsolateral striatum (DLS) as rats performed two reward-guided choice tasks, in which we manipulated either the size of or delay to reward. During these tasks, rats first learned which of two options led to the better reward and then reversed those contingencies in a second block of trials. We found that rats with HDAC5 overexpression in DS responded faster and chose higher value reward more often during the first block of trials but were less able to reverse those contingencies in the second block of trials. At the neural level, HDAC5 overexpression in DS elevated and reduced the number of cells in DLS that increased firing to stimuli and reward, respectively, and shifted encoding toward cues that predicted more immediate reward. These results suggest that the HDAC5 overexpression in DS contributes to inflexible decision-making, demonstrating a role of histone-modifying enzymes in complex cognitive function.

**SIGNIFICANCE STATEMENT** HDACs are important for learning and habit formation. Here, we expanded on these functions and found that overexpression of HDAC5 produced faster and more automatic behavior, and related changes in dorsolateral striatal neural firing in rats performing a value-based decision-making task. These results implicate HDAC5 as a potential therapeutic target for psychiatric conditions that impair decision-making and executive function.

## Introduction

Histone deacetylases (HDACs) are a family of epigenetic enzymes that suppress gene transcription by removing acetyl groups from histone proteins (Kouzarides, 2007). Over the past decade, extensive literature has demonstrated that HDACs, primarily class I HDACs (HDAC1, HDAC2, and HDAC3), contribute to synaptic plasticity associated with learning and memory (Peixoto and Abel, 2013; Mahgoub and Monteggia, 2014; Penney and Tsai, 2014; Schmauss, 2017). For example, overexpressing HDAC2 in mouse hippocampus disrupts synaptogenesis, synaptic formation, long-term potentiation, and hippocampal-dependent learning and memory formation, whereas opposite effects are observed in HDAC2-deficient mice (Guan et al., 2009). Emerging evidence showed that HDAC3 in rat dorsal striatum (DS) also regulates associative learning, such as habit formation (Malvaez et al., 2018). However, whether HDACs contribute to complex executive cognitive functions, such as reward-guided decision-making, is unknown. More intriguingly, how epigenetics influence neural activity at the single-cell level during decision-making tasks has not been explored. Dysregulated HDAC function has been linked to aging, neurodegenerative diseases (e.g., Alzheimer's disease), and psychiatric disorders (e.g., drug addiction; Peña et al., 2014; Hwang et al., 2017; Schmauss, 2017; Werner et al., 2021). Therefore, elucidating the role of HDACs in executive cognitive functions can help in understanding how dysregulated HDAC activity may lead to impaired cognitive functions under these pathologic conditions (Forman et al., 2004; Volkow and Li, 2004; Koob and Volkow, 2010; Samson and Barnes, 2013; Murman, 2015; Wyss-Coray, 2016).

Here, we examined the role of HDAC5, a class IIa HDAC, in rat DS in reward-guided decision-making and associated neural encoding. Like other class IIa HDACs (HDAC4, HDAC7, and HDAC9), HDAC5 can shuttle into the nucleus from cytoplasm on dephosphorylation in an activity-dependent manner (McKinsey et al., 2000; Borrelli et al., 2008). We focused on striatal HDAC5 based on its critical role in regulating drug-related behaviors in rodent models. For example, HDAC5 and its downstream targets in nucleus accumbens (NAc) modulate the rewarding aspect of cocaine (McKinsey et al., 2000; Renthal et al., 2007; Borrelli et al., 2008; Taniguchi et al., 2012, 2017). Work from us and others also demonstrates that HDAC5 in NAc and DS contributes to cocaine and methamphetamine relapse, respectively (Taniguchi et al., 2017; Li et al., 2018). Such findings suggest that the study of HDAC5 here can provide important insight into our observations that behavioral deficits in decision-making develop after chronic drug use (Stalnaker et al., 2006, 2009; Roesch et al., 2007; Takahashi et al., 2007; Burton et al., 2015, 2017, 2018; Brockett et al., 2018; Vázquez et al., 2019; Pribut et al., 2021).

To examine the role of HDAC5 in value-based decision-making behavior, we overexpressed HDAC5 in DS. We additionally recorded from dorsolateral striatum (DLS), a region known for its well-established involvement in forming and governing habits by encoding associative information among stimuli, outcomes, and responses (e.g., stimulus–response associations; Yin and Knowlton, 2006; Balleine et al., 2007; Burton et al., 2015, 2017; Malvaez and Wassum, 2018). Our results showed that HDAC5 overexpression in DS promoted fast, inflexible behavior during reward-guided decision-making, accompanied by enhanced and reduced firing to cues and reward in DLS, respectively. These findings provide the first evidence, to our knowledge, that HDAC5 in DS contributes to impulsive and inflexible decision-making, behavioral deficits previously observed after chronic drug use (Stalnaker et al., 2006, 2009; Roesch et al., 2007; Takahashi et al., 2007; Burton et al., 2015, 2017, 2018; Brockett et al., 2018; Vázquez et al., 2019; Pribut et al., 2021). Therefore, HDAC5 in striatum may serve as a candidate epigenetic target that underlies the impaired cognition associated with drug addiction.

## Materials and Methods

### Subjects

Eighteen Sprague Dawley rats, both male and female, were obtained at 175–200 g from Charles River Laboratories. Seven rats were excluded because of death during surgeries (*n* = 1) and electrode implantation issues (*n* = 6). The remaining 11 rats (9 males, 2 females) refer hereafter to those used in statistical analyses. Subjects were tested at the University of Maryland, College Park in accordance with university and National Institutes of Health guidelines.

### Reward-guided choice tasks

**Figure 1.**
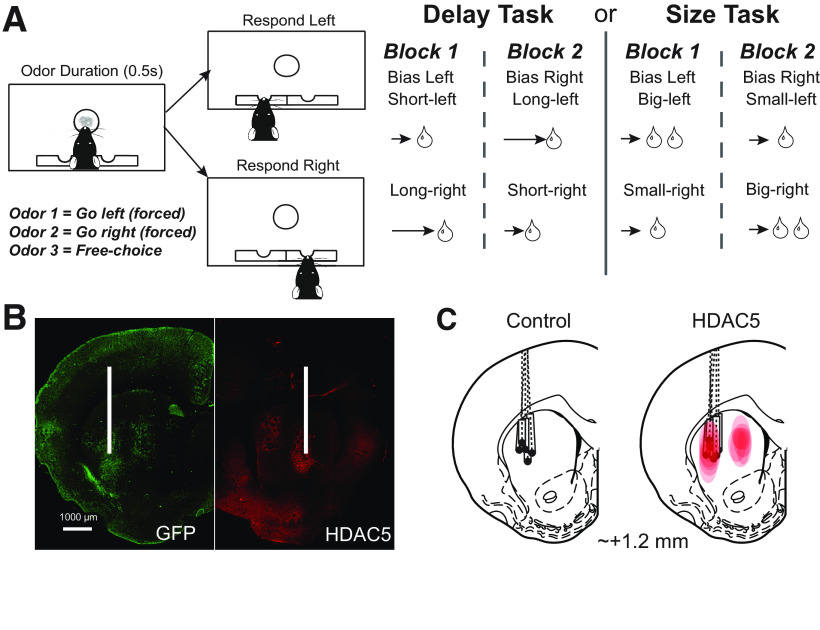
Reward-guided decision-making task, virus injections/expressions, and recording sites. ***A***, Schematics of the reward-guided decision-making task, showing the sequence of events in a single trial (left) and the sequence of blocks for delay and size tasks. ***B***, Representative images of GFP expression from control rats and HDAC5 immunostaining from HDAC5 group. White lines indicate placement of electrodes. ***C***, Electrode placements in control and HDAC5 rats, and virus spread in HDAC5 rats (right diagram, red area). Note the overlap between the recording area and virus expression in HDAC5 rats.

Before surgery, the rats were trained on the delay/size choice task (Fig. 1*A*) for ∼1 month. During the task, rats were trained to nose poke into the central odor port on illumination of the house light. While in the odor port, the rat was exposed to one of three odor cues (2-Octanol, pentyl acetate, or carvone) that directed the rat on how to obtain the 10% sucrose water reward. Two odor cues were forced choices, where the rat was instructed to go to either the left or right well to receive reward. The third cue was a free choice, where the rat would be rewarded at either well. The cues associated with each odor were maintained across sessions. Odors were counterbalanced across rats and presented in a pseudorandom sequence with equal distributions of left/right odors and free-choice odor occurring on 7/20 trials. Rats were water deprived to encourage motivation in the tasks. If the incorrect well was selected on a forced-choice trial, the houselights turned off, and no reward was delivered. During initial training, rats were first trained to nose poke and then go to the wells for immediate delivery of reward for 1–2 d. After that, we introduced free-choice trials, where rats could choose one well or the other to obtain reward. Then, we gradually introduced (2 per day) forced-choice trials. While training them on these contingencies, we progressively increased (100 ms per day) how long rats had to stay in the odor port and fluid wells until they could remain in the odor port for 1 s and wait for delayed rewards up to 7 s.

Rats underwent one recording session per day and alternated between two session types: delay and size blocks. At the start of delay sessions, one well was randomly designated to have short delays (500 ms) and the other to have long delays (1000–7000 ms) before reward delivery. On long-delay trials, the delay increased by 1000 ms each time the long-delay well was chosen by the rat during a free-choice trial. The maximum delay time was 7000 ms and could be reduced by 1000 ms on each free-choice trial if the long-delay well had been chosen <8 times of the last 10 trials to a minimum delay of 3000 ms. After 60 trials, short- and long-delay parameters were switched to opposite wells. At the start of size sessions, one well was randomly designated as the big well and the other as the small well. The small well delivered one 0.05 ml 10% sucrose water bolus, and the big well delivered two 0.05 ml 10% sucrose water boluses with the second one appearing 500 ms after the first. The delay time before reward delivery was held constant throughout the session at 500 ms. After 60 trials, the big and small parameters were switched to the opposite wells.

### Adeno-associated virus injections

We used adeno-associated virus serotype 2 (AAV2), which shows mostly neuronal tropism (Haery et al., 2019). Both AAV2-mHDAC5 (5 × 10^e12^ viral particles/ml) and AAV2-GFP (4 × 10^e12^ viral particles/ml) are driven by CMV promoters. AAV2-mHDAC5 expressed a dephosphorylated mutant of HDAC5 (S259A/S279A/S498A or 3SA) that was primarily localized to the nucleus (Taniguchi et al., 2017). AAV2-GFP expressed green fluorescence proteins and was used as the control condition. Detailed plasmid maps are available on request. The *in vivo* validation of HDAC5 overexpression by AAV2-mHDAC5 and comparisons to baseline expression of HDAC5 were previously demonstrated by Li et al. (2018).

Rats received either bilateral AAV2-mHDAC5 (*n* = 6, five males, one female) or AAV2-GFP injections (*n* = 5, four males, one female) as described previously (Li et al., 2018). Briefly, each hemisphere received a total of four injections (0.75 μl/injection), with two injections aiming at the dorsomedial striatum (DMS) and the other two injections aiming at DLS. We used the following coordinates from bregma: for DMS, anteroposterior (AP): +1.2 mm, medial lateral (ML): ±2.6 mm (6° angle), dorsal ventral (DV): −4.0 mm and −5.0 mm; for DLS, AP: +1.2 mm, ML: ±3.8 mm (6° angle), DV: −5.0 mm and −6.0 mm. We delivered the AAVs by Hamilton syringes (32 gauge) at a rate of 0.375 μl/min. After each injection, we left the injection needle in place for an additional minute to allow diffusion. After the final injection, we filled the drilled hole with bone wax. It is noted that we overexpressed HDAC5 in the entire DS. This is based on previous observations that HDAC5 manipulations need to be administered to the entire DS as those targeting the DLS or DMS alone are ineffective in modulating drug seeking (Li et al., 2018).

### Electrode implantation

Immediately after virus injections, we implanted unilateral drivable electrodes (bundles of 10- to 25-µm-diameter FeNiCr wires, cut at an angle so that wires are at different lengths) in the DLS (AP: +1.2 mm, ML: 3.2 mm, DV: −3.5 mm) for subsequent single-unit recordings, and we counterbalanced the hemispheres of the electrode implantations (Fig. 1*C*).

### Single-unit recording

Procedures were the same as described previously (Bryden and Roesch, 2015). Wires were screened for activity daily; if no activity was detected, the rat was removed, and the electrode was advanced 40 or 80 µm to reach new cells. Otherwise, a session was conducted, and the electrode was advanced at the end of the session. Neural activity was recorded using four identical Plexon Multichannel Acquisition Processor systems. Signals from electrode wires were amplified 20× by an operational amplifier headstage, located on the electrode array. Immediately outside the training chamber, the signals were passed through a differential preamplifier (PBX2/16sp-r-G50/16fp-G50, Plexon), where single-unit signals were amplified 50× and filtered at 150–9000 Hz. Single-unit signals were then sent to the Multichannel Acquisition Processor box, where they were further filtered at 250–8000 Hz, digitized at 40 kHz, and amplified at 1–32×. Waveforms (>2.5:1 signal-to-noise) were extracted from active channels and recorded to disk by an associated workstation with event time stamps from the behavior computer.

### Experimental design and statistical analyses

Behavior during the reward-guided choice tasks was analyzed by calculating percentage choice of a particular valued condition (i.e., short, long, large, small) on free-choice trials, reaction times on free-choice trials (i.e., odor offset to odor port exit), and movement time on free-choice trials (i.e., odor port exit to fluid well entry). Like previous studies (Burton et al., 2017, 2018; Brockett et al., 2018; Vázquez et al., 2019; Pribut et al., 2021), percent choice analyses included the first and last 10 trials in respective reward categories to examine changes in behavior after a block switch. We additionally examined behavior by session day to determine whether there were any transient changes to behavior during the recording period (Vaidya et al., 2019).

Behavioral analyses were computed for each individual session and averaged across sessions for HDAC5 and control groups. We took the minimal number of sessions collected from one rat and then used that same number of sessions for each rat split over the entirety of recording. Thus, each rat contributed the same number of sessions to the behavioral analyses. For reaction and movement times, we used a repeated-measures ANOVA test with factors for group (Control vs HDAC5), task (delay vs size), and session day as a within-subjects measure. For percent choice, we used a repeated-measures ANOVA test with factors of group (Control vs HDAC5), phase (first vs last 10 trials), block (first or second), and session day as a within-subjects measure. Bonferroni-corrected *post hoc t* tests were used to further examine significant interaction terms.

Single units were sorted using template matching software in Offline Sorter (Plexon). Time stamps and event markers were extracted from the file using NeuroExplorer (Nex Technologies). Data were analyzed using RStudio and MATLAB (MathWorks). Analysis epochs were calculated by taking the total number of spikes and dividing by time. Baseline firing activity was taken 1 s before odor onset. Increasing- and decreasing-type neurons were designated based on whether firing increased or decreased significantly relative to the baseline (Wilcoxon; *p* < 0.05). The odor cue epoch was taken 100 ms after odor onset until well entry. The reward epoch encompassed 250 ms before sucrose delivery to 1 s after reward delivery. This epoch has been used previously (Burton et al., 2017, 2018; Vázquez et al., 2019; Pribut et al., 2021) to capture firing related to the anticipation and delivery of reward. The epoch captures activity immediately preceding reward delivery without overlapping with movement-related firing even at the shortest delays (i.e., 500 ms) and captures firing related to the multiple sucrose boluses delivered during large reward trials. Relationships between neural firing and behavioral activity were determined with regression tests for each neuron separately. Specifically, regressions were performed on trial firing rates and reaction times collected during each recording session, as opposed to averaging across trials.

### Histology

Rats were deeply anesthetized with isoflurane and perfused transcardially with 500 ml of 0.01 m PBS. Brain tissue was then fixed with 500 ml of 4% paraformaldehyde (PFA) for 1 h before being transferred into 30% sucrose PBS solution. Once the brains sank, they were sectioned into 30 μm slices using a Leica cryostat and stored in cryoprotectant at −80°C. For HDAC5 immunohistochemistry, the sections were washed for 10 min in PBS and then incubated for 1 h at room temperature in blocking buffer (2% BSA in PBS with 0.3% Triton X-100). The sections were incubated next with a primary antibody against HDAC5 (1:500; catalog #sc-133106, Santa Cruz Biotechnology; RRID:AB_2116793) in blocking buffer overnight at room temperature. After washing the sections three times in PBS (5 min each), they were incubated with the secondary antibody Alexa 594-labeled anti-mouse (1:200; catalog #R37121, Thermo Fisher Scientific; RRID:AB_2556549) in blocking buffer for 1 h at room temperature. Finally, the sections were washed in PBS and mounted on glass slides (Fisherbrand Superfrost Plus Microscope Slides, catalog #12-550-15, Fisher Scientific) that were air dried and cover slipped with Fluormount G (Electron Microscopy Sciences).

### Data availability

Raw data and MATLAB codes used for behavioral and neural analyses are available on request.

## Results

### HDAC5 overexpression in DS decreased reaction time during both delay and size tasks

**Figure 2.**
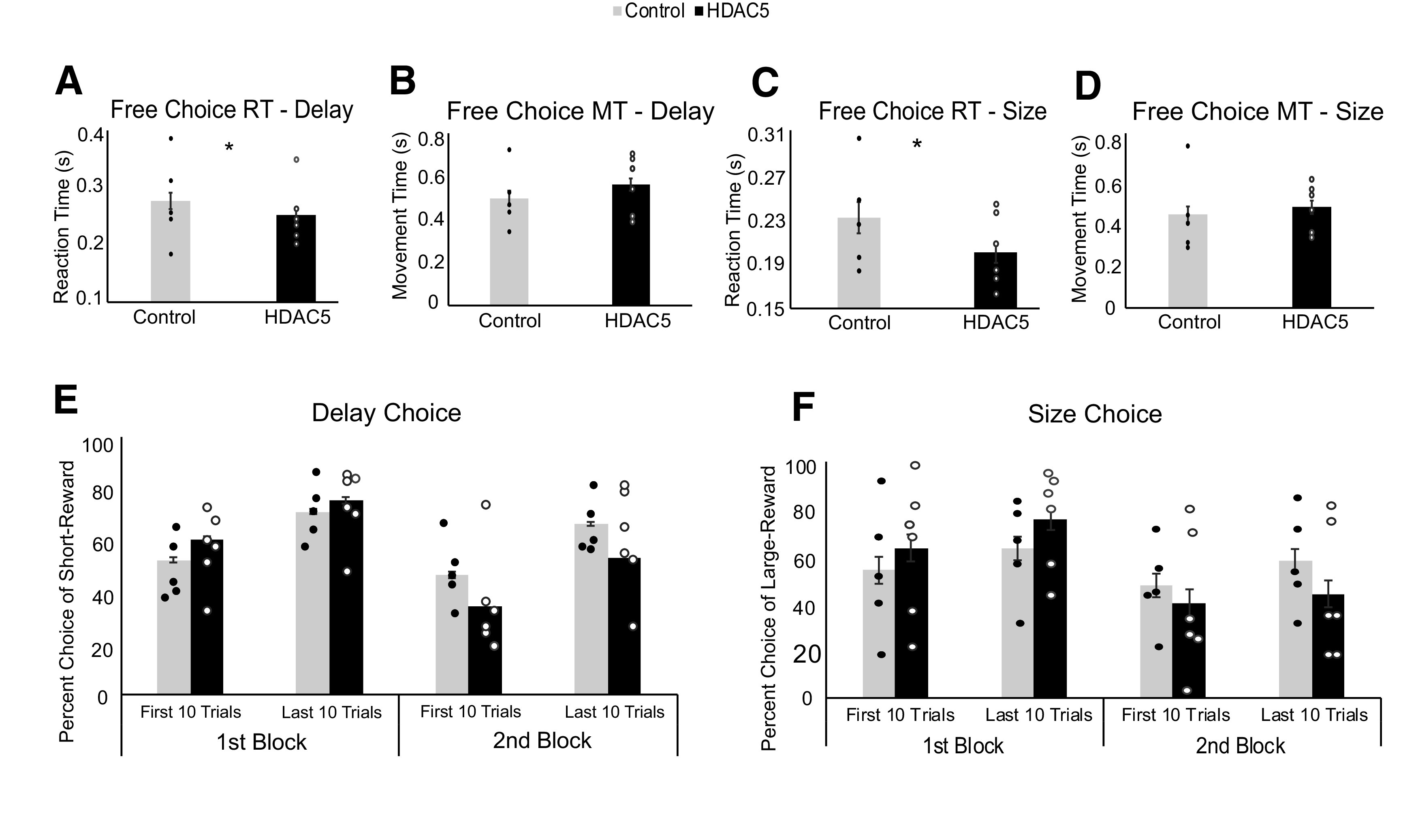
HDAC5 overexpression decreased reaction time and made choice behavior less flexible. Dots represent individual rat averages. Asterisks indicate significance. ***A***, Reaction time (RT; odor offset to odor port exit) averaged over all correct trials and recording sessions. We took the minimal number of sessions collected from one rat and used that same number of sessions for each rat split over the entirety of recording for the delay task (Control rats, *n* = 162, 123, 111, 49, 40; HDAC5 rats, *n* = 153, 132, 118, 63, 36, 35). ***B***, Movement time (MT; odor port exit to fluid well entry). ***C***, ***D***, Same as ***A*** and ***B*** during performance of the size task (Control rats, *n* = 103, 98, 63, 46, 45; HDAC5 rats, *n* = 143, 114, 99, 61, 33, 28). ***E***, Percent choice of short-delay trials during blocks 1 and 2. Within each block, trials were split into first and last 10 free-choice trials. *Post hoc t* tests for significant interactions between group and block were averaged across these early and late periods of a session. ***F***, Same as ***E*** during performance of the size task.

Our first analyses examined reaction time, defined as the time taken to exit the central nose port after presentation of the odor stimulus, across delay (8 d/rat) and size (6 d/rat) tasks (Fig. 2*A*,*C*). We analyzed data using a repeated-measures ANOVA with factors of group (Control vs HDAC5), task (delay vs size), and session day. A significant main effect of task demonstrated that all rats exhibited significantly faster reaction times during size tasks (*F*_(1,146)_ = 11.005, *p* = 0.001, ANOVA). Furthermore, a significant main effect of group showed that HDAC5 rats were also faster overall across both task manipulations (*F*_(1,146)_ = 4.345, *p* = 0.039, ANOVA). There was no significant effect of session day (*F*_(1,146)_ < 0.01, *p* > 0.05, ANOVA), nor were there any significant interactions (*F*_(1,146)_ ≤ 1.734, *p* ≥ 0.881, ANOVA).

Based on the assumption that rats leave the odor port once they have made their choice selection, one interpretation of the reaction time result is that rats with HDAC5 overexpression are making faster decisions than control rats. However, an alternative interpretation is that HDAC5 rats simply exhibit enhanced motor responses in general. To address this issue, we also measured movement time as defined as the time from odor port exit to the fluid well as a general reflection of movement speed (Fig. 2*B*,*D*). We found no significant main effects of group (*F*_(1,146)_ = 2.426, *p* = 0.122, ANOVA), task (*F*_(1,146)_ = 0.989, *p* = 0.322, ANOVA), or session day (*F*_(1,146)_ < 0.01, *p* > 0.05), nor were there any significant interactions (*F*_(1.146)_ ≤ 2.426, *p* ≥ 0.122, ANOVA). Thus, HDAC5 overexpression selectively decreased reaction time but caused no general motor enhancement.

### HDAC5 overexpression in DS promoted inflexible behavior

Decisions governed under habitual control are thought to be under the control of model-free systems that do not take into account task structures (e.g., frequent reversals), thus allowing animals to respond without deliberations and to develop associative behaviors more strongly and quickly. There is a trade-off, however, with behavioral flexibility. In line with these theories, we found that HDAC5 rats formed stronger associations in the first block of trials that were difficult to reverse in the second block of trials.

Our next set of analyses (Fig. 2*E*,*F*) examined the percent choice on free-choice trials for high-value choices (i.e., short delay or large reward), broken down into first or last 10 trials for blocks 1 and 2 (Note, there were ∼20 free-choice trials per block that were randomly interleaved with forced-choice trials.). We used a repeated-measures ANOVA to analyze these data with factors of group (Control vs HDAC5), task (delay vs size), phase (first 10 vs last 10 trials within a block), block (first vs second) and session day.

For both delay (Fig. 2*E*) and size tasks (Fig. 2*F*), we observed a significant main effect of phase (*F*_(1,584)_ = 45.543, *p* < 0.001, ANOVA), indicating that all rats selected high-value rewards significantly more during the end of a trial block compared with the beginning. A main effect of block additionally showed all rats selected high-value rewards significantly more in the first compared with the second block of trials (*F*_(1,584)_ = 35.161, *p* < 0.001, ANOVA), likely because rats were overriding previously learned reward contingencies they had acquired in the first block. There was no significant main effect of session day (*F*_(1,584)_ < 0.01, *p* > 0.05, ANOVA) or task (*F*_(1,584)_ = 0.004, *p* = 0.950, ANOVA). There was significant interaction between task and phase (*F*_(1,584)_ = 5.724, *p* = 0.017, ANOVA), however Bonferroni-corrected *post hoc t* tests indicated no significant differences between either the first 10 trials of delay and size tasks (*t*_(306)_ = −1.176, *p* = 0.240, *t* test), nor the last 10 trials of delay and size tasks (*t*_(306)_ = 2.172, *p* = 0.031, *t* test). Thus, all rats generally chose more high-value rewards by the last 10 trials and during the first block, and there were no significant differences between tasks.

Interestingly, although there was no significant main effect of group (*F*_(1,584_) = 0.063, *p* = 0.803, ANOVA), we did observe a significant interaction between group and block (*F*_(1,584_) = 12.745, *p* < 0.001, ANOVA). Bonferroni-corrected *post hoc t* tests indicated that HDAC5 rats selected high-value rewards significantly more than control rats during block 1 (*t*_(306)_ = 2.366, *p* = 0.019, *t* test), but subsequently selected high-value rewards significantly less than control rats during block 2 (*t*_(306)_ = −2.743, *p* = 0.006, *t* test). All other interactions were not significant (*F*_(1,584)_ < 0.001, *p* > 0.05). Together, these results suggest that rats generally performed best by the end of the first block and needed to reverse reward contingencies in the second block. This contrast in performance between the first and second block was significantly amplified by HDAC5 overexpression, across both delay and size tasks.

### HDAC5 overexpression in DS elevated the number of neurons that increased firing to reward predicting stimuli

**Figure 3.**
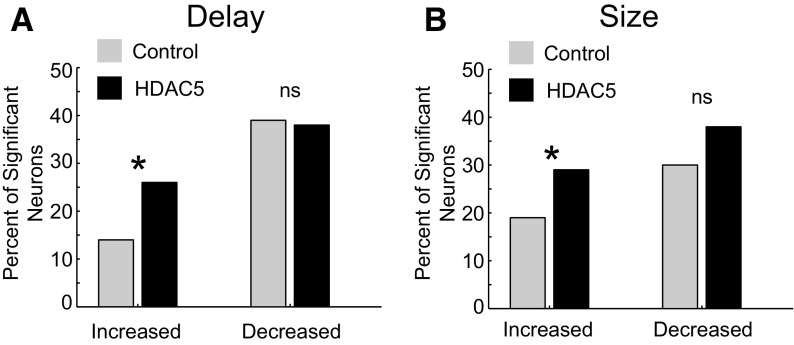
HDAC5 overexpression elevated the count of neurons that increased firing during stimulus presentation. Asterisks indicate significance. ***A***, ***B***, Percent of cells that significantly increased or decreased firing during odor sampling (odor onset to port exit) compared with baseline (1 s epoch starting 1 s before odor onset; Wilcoxon, *p* < 0.05) during the delay (Control: 14% increased, 39% decreased; HDAC5: 26% increased, 38% decreased; ***A***) and size (Control: 19% increased, 29% decreased; HDAC5: 30% increased, 38% decreased; ***B***) tasks.

During the delay task, we recorded from the DLS, 485 neurons in control rats (*n* = 162, 123, 111, 49, 40) and 537 neurons in HDAC5 rats (*n* = 153, 132, 118, 63, 36, 35). In control rats, 14% (*n* = 66; 31, 17, 10, 7, 1) and 39% (*n* = 189; 89, 54, 17, 15, 14) of neurons significantly increased and decreased firing during odor cue sampling, respectively (Fig. 3*A*, gray). In HDAC5 rats, 26% (*n* = 137; 83, 14, 14, 12, 10, 4) and 38% (*n* = 205; 88, 56, 28, 16, 9, 8) of neurons significantly increased and decreased firing, respectively (Fig. 3*A*, black). The frequency of increasing to decreasing neurons was significantly higher in HDAC5 rats (Χ^2^ = 12.5; *p* = 0.0004, Χ^2^) as was the frequency of increasing to total cells recorded (Χ^2^ = 14.7; *p* = 0.0001, Χ^2^). The counts of decreasing cells did not significantly differ between groups (Χ^2^ = 0.01; *p* = 0.91, Χ^2^). We also found no significant difference in baseline activity in either increasing (*t*_(171)_ = −0.002, *p* = 0.999, unpaired *t* test) or decreasing cells (*t*_(392_) = 0.250, *p* = 0.802, unpaired *t* test).

During the size task, we recorded from the DLS 355 neurons in control rats (*n* = 103, 98, 63, 46, 45) and 478 neurons in HDAC5 rats (*n* = 143, 114, 99, 61, 33, 28). In control rats, 19% (*n* = 67; 38, 10, 8, 6, 5) and 29% (*n* = 102; 44, 26, 15, 9, 8) of neurons significantly increased and decreased firing during odor cue sampling, respectively (Fig. 3*B*, gray). In HDAC5 rats, 30% (*n* = 142; 88, 18, 13, 10, 9, 4) and 38% (*n* = 181; 92, 39, 27,9, 8, 6) of neurons increased and decreased firing, respectively (Fig. 3*B*, black). The frequency of increasing to total cells recorded was significantly higher in HDAC5 rats (Χ^2^ = 7.3; *p* = 0.007, Χ^2^) than control rats, whereas the frequency of decreasing cells did not significantly differ between groups (Χ^2^ = 3.5; *p* = 0.06, Χ^2^). We found no significant difference in baseline activity in either increasing (*t*_(203)_ = −1.273, *p* = 0.204, unpaired *t* test) or decreasing cells (*t*_(159)_ = 1.246, *p* = 0.215, unpaired *t* test). Together, overexpressing HDAC5 in DS elevated the counts of DLS neurons that increased firing during sampling of reward-predicting stimuli across tasks.

### HDAC5 overexpression in DS increased the number of neurons that fired more strongly for short-delay reward

**Figure 4.**
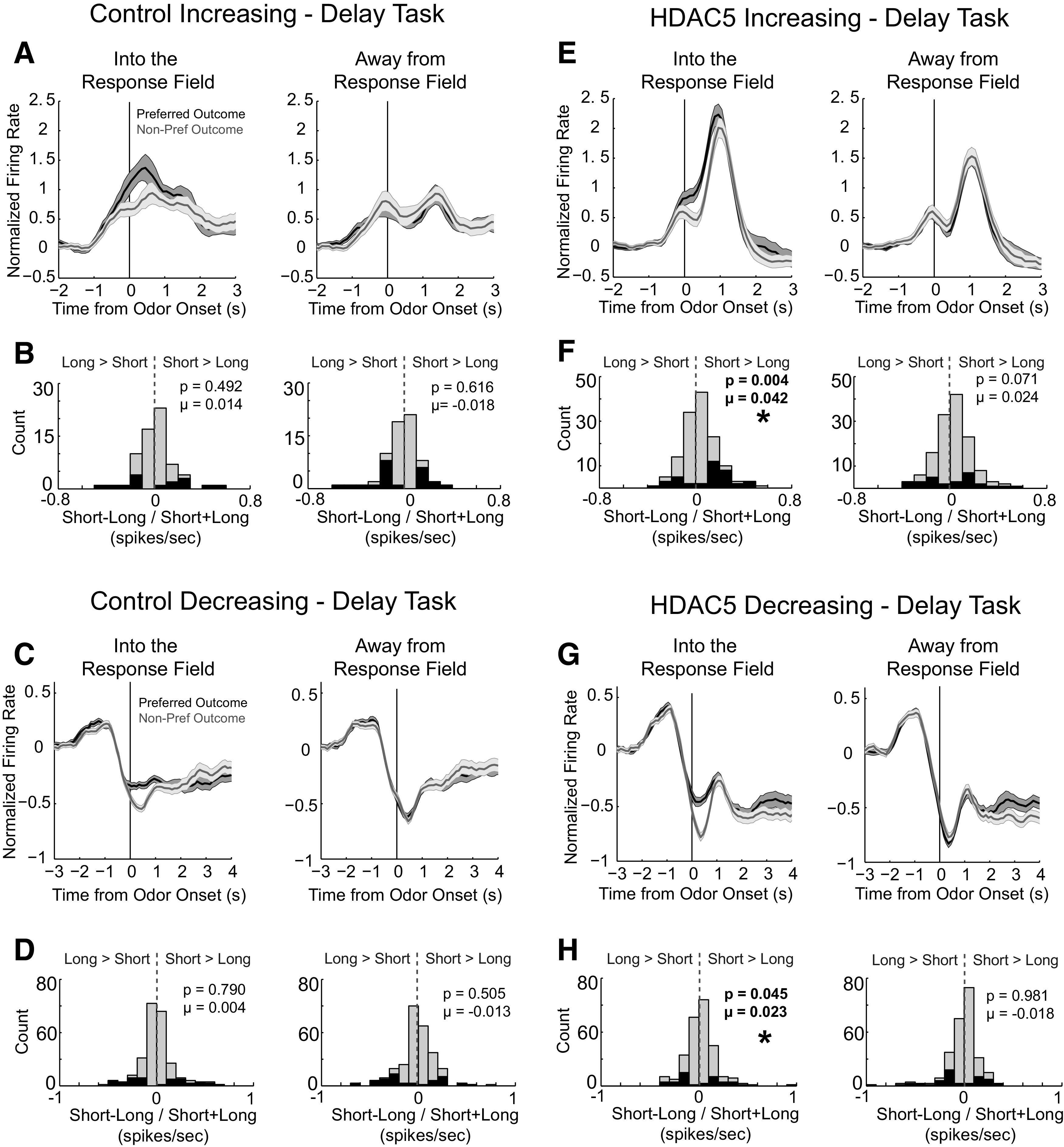
HDAC5 overexpression increased movement-related firing and the proportion of cells firing to short-delay predicting cues. Asterisks indicate significance. ***A***, Average firing over all control cells that increased firing during odor sampling (increasing, *n* = 66; decreasing, *n* = 189) aligned to odor onset. We sorted firing into preferred (i.e., toward the response field of the neuron) and nonpreferred directions (i.e., away from the response field of the neuron), and preferred and nonpreferred outcomes, based on the direction and outcome that elicited the highest firing rate. ***B***, Distribution of delay indices computed for neuron during the odor epoch (short–long/short+long). Gray bars reflect the distribution of indices across the entire population of neurons, and black bars represent neurons that exhibit a preference for short- or long-delay trials, firing significantly more (above 0; short > long) or firing significantly less (below 0; long > short) for cues that predicted short delayed reward. ***C***, ***D***, Same as ***A*** and ***B*** except for cells that decreased firing during the odor epoch (*n* = 189). ***E–H***, Same as ***A–D*** with data from HDAC5 rats (increasing, *n* = 137; decreasing, *n* = 205).

Previously, we have shown that neurons in DLS will fire more strongly for certain directions (e.g., left or right), and for certain outcomes (e.g., short, long, big, or small) with similar distributions of each. Here, we examined temporal firing of increasing and decreasing neurons and quantified this selectivity during cue sampling. We sorted firing into preferred (i.e., toward the response field of the neuron; Fig. 4, left) and nonpreferred directions (i.e., away from the response field of the neuron; Fig. 4, right) and preferred and nonpreferred outcomes, based on the direction and outcome that elicited the highest firing rate. For example, if a neuron fired the strongest to cues that predicted short delayed reward for responses made in the left direction, short delayed reward was designated as the preferred outcome and left was designated as the response made into the response field of that cell. In this example, long delayed reward would be the nonpreferred outcome, and right would be the response made away from the response field. This procedure simply allows us to average firing across all neurons so that we can examine temporal changes in firing to task events.

Figure 4, *A* and *E*, illustrates normalized firing—aligned to odor onset—for neurons that increased firing in control and HDAC5 rats during delay blocks. Data were normalized by *z*-scoring. As defined, increases in firing were present during odor sampling. Interestingly, although firing in control rats peaked near the end of odor sampling (500 ms after onset) before the onset of the movement, firing in HDAC5 rats exhibited a more dramatic rise after odor presentation (>500 ms after odor onset) during initiation of the movement, which likely reflected accelerated responses as described above (Fig. 2) and further analyzed below by showing correlations between firing rate and reaction time at the level of single neurons.

To quantify selectivity during the delay task, we plotted the normalized difference between short and long (delay index = short – long/short + long) for each neuron for control and HDAC5 rats. Gray bars reflect the distribution of indices across the entire population of neurons, and black bars represent neurons that exhibit a preference for short- or long-delay trials, firing significantly more (above zero; short > long) or firing significantly less (below zero; long > short) for cues that predicted short delayed reward (Fig. 4*B*). Examining activity across odor-responsive cells from control rats, we found distributions of delay indices were not shifted significantly above or below zero (into: *p* = 0.492, µ = 0.014, Wilcoxon; Fig. 4*B*, left; away: *p* = 0.616, µ = −0.018, Wilcoxon; Fig. 4*B*, right). Interestingly, in HDAC5 rats, the preference for neurons to increase firing to short delayed reward increased. Unlike controls, the distribution of delay indices in HDAC5 rats was significantly shifted above zero for movements made into the response field (*p* = 0.004, µ = 0.042, Wilcoxon; Fig. 4*F*, left), indicating that the majority of neurons tended to fire more strongly for cues that predicted short delayed reward. Similar results were present for neurons that decreased firing during odor presentation (i.e., Fig. 4*H*, left; *p* = 0.045, µ = 0.023, Wilcoxon).

**Figure 5.**
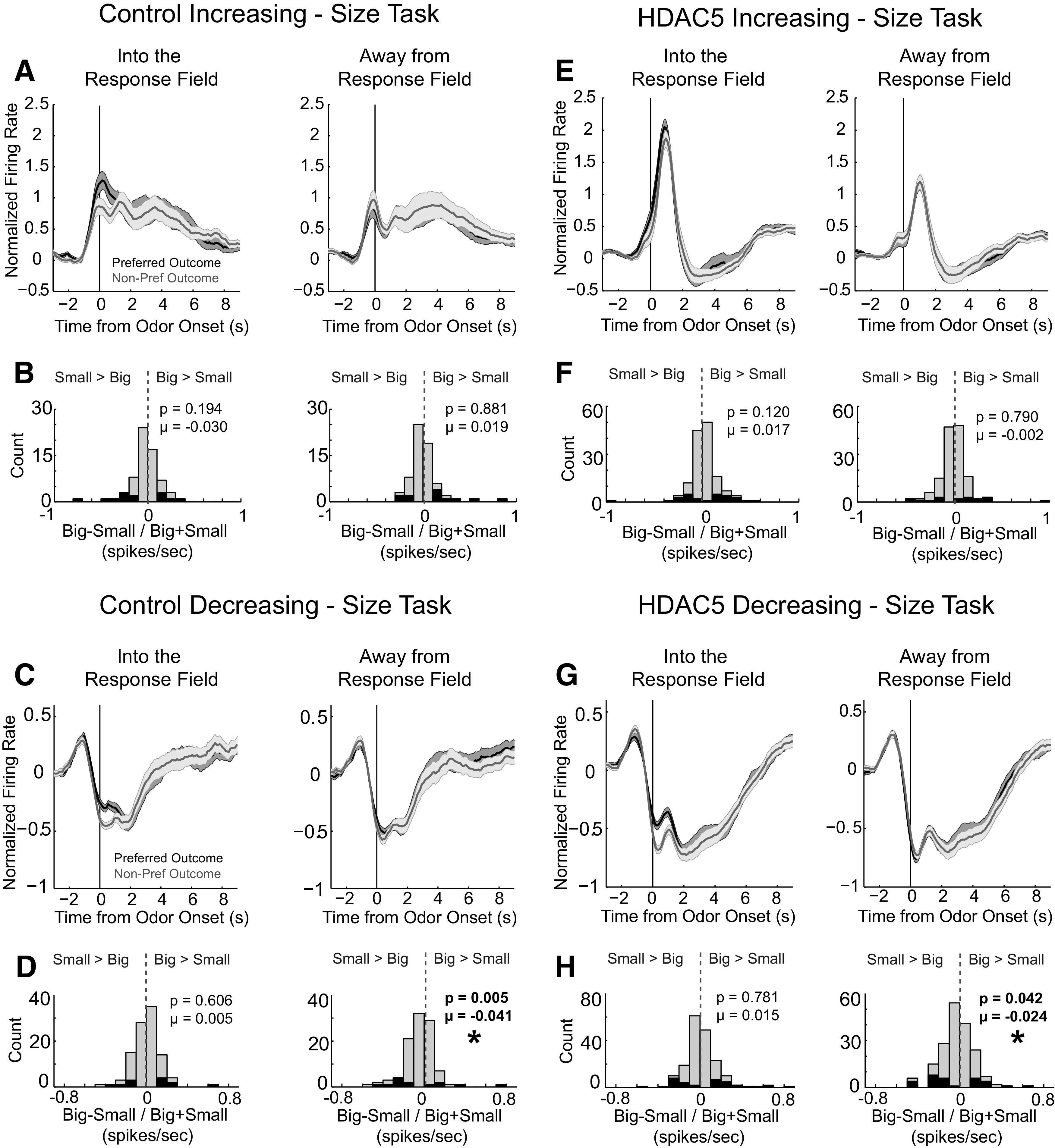
HDAC5 overexpression in dorsal striatum increased movement-related firing during the size task. Asterisks indicate significance. ***A***, Average firing over all control cells that increased firing (increasing, *n* = 67; decreasing, *n* = 102) during odor sampling aligned to odor onset. We sorted firing into preferred (i.e., toward the response field of the neuron) and nonpreferred directions (i.e., away from the response field of the neuron), and preferred and nonpreferred outcomes, based on the direction and outcome that elicited the highest firing rate. ***B***, Distribution of size indices computed for neurons during the odor epoch (large–small/large+small). Gray bars reflect the distribution of indices across the entire population of neurons, and black bars represent cells with significant differences between large and small (Wilcoxon; *p* < 0.05). ***C***, ***D***, Same as ***A*** and ***B*** except for cells that decreased firing during the odor epoch (*n* = 102). ***E–H***, Same as ***A–D*** with data from HDAC5 rats (increasing, *n* = 142; decreasing, *n* = 181).

These analyses were repeated for neurons responsive to odor cues during size blocks (Fig. 5). Across conditions, response profiles were similar to those observed during delay blocks; however, unlike delay manipulations, shifts in selectivity distributions (size index = large – small/large + small) did not differ between groups, suggesting the size encoding was not altered by HDAC5 overexpression beyond there being fewer task-related neurons overall. For increasing-type cells, none of the distributions, for either control or HDAC5 rats, were significantly shifted (Fig. 5*B*, into: *p* = 0.194, µ = −0.030, Wilcoxon, left; away: *p* = 0.881, µ = 0.019, Wilcoxon, right; Fig. 5*F*, into: *p* = 0.120, µ = 0.017, Wilcoxon, left; away: *p* = 0.790, µ = −0.002, Wilcoxon, right). This was also observed in neurons that decreased firing for responses made into the response field (Fig. 5*D*, Control, left: *p* = 0.606, µ = 0.005, Wilcoxon; Fig. 5*H*, HDAC5, left: *p* = 0.781, µ = 0.015, Wilcoxon). For responses made away from the response field, distributions in both groups were shifted in the negative direction (Fig. 5*D*, right; *p* = 0.005, µ = −0.041, Wilcoxon; Fig. 5*H*, right; *p* = 0.042, µ = −0.024, Wilcoxon).

### Firing of single neurons was correlated with reaction time

**Figure 6.**
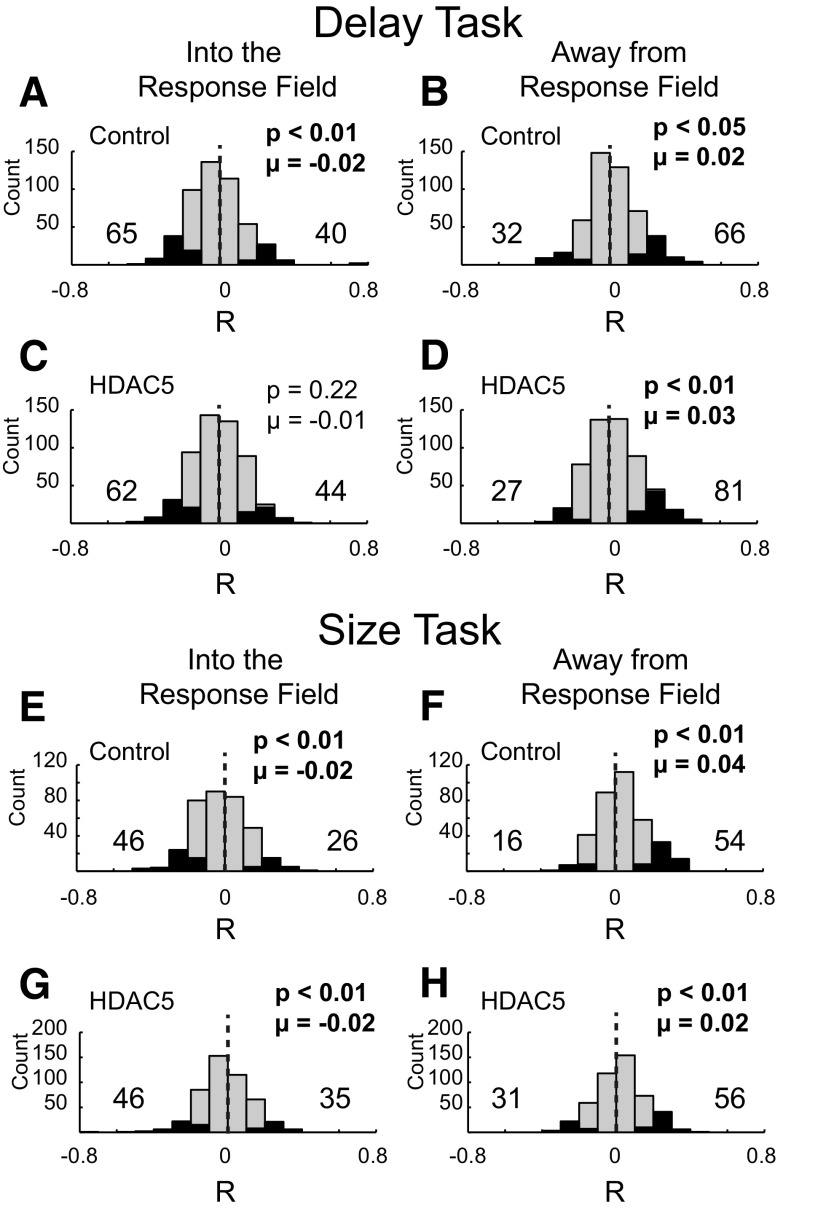
Firing in dorsolateral striatum was correlated with reaction time. ***A***–***D***, Distribution of R values for within-cell correlations between firing rate and reaction time in control (***A***, ***B***) and HDAC5 (***C***, ***D***) rats for movements made into (***A***, ***C***, left) and away from (***B***, ***D***, right) from the response field during the delay task. ***E***–***H***, same as ***A***–***D***, but during the size task.

To better understand the relationship between behavior and changes in firing, we examined the correlation between firing rate and reaction time for each single neuron. Overall, firing tended to be negatively correlated with reaction time for movement made into the response field of a cell and positively correlated for movements made away from the response field of a cell, suggesting that increases and decreases in firing promoted and attenuated choices to be made into the response field of the cell, respectively. For movement into the response field of the cell, all distributions were shifted in the negative direction (Control delay: Fig. 6*A*, *p* < 0.01, µ = −0.02, Wilcoxon; HDAC5 delay: Fig. 6*C*, *p* < 0.22, µ = −0.01, Wilcoxon; Control size: Fig. 6*E*, *p* < 0.01, µ = −0.02, Wilcoxon; HDAC5 size: Fig. 6*G*, *p* < 0.01, µ = −0.02, Wilcoxon), whereas for movement away from the field of the cell, all distributions were shifted in the positive direction (Control delay: Fig. 6*B*, *p* < 0.05, µ = 0.02, Wilcoxon; HDAC5 delay: Fig. 6*D*, *p* < 0.01, µ = 0.03, Wilcoxon; Control size: Fig. 6*F*, *p* < 0.01, µ = 0.04, Wilcoxon; HDAC5 size: Fig. 6*H*, *p* < 0.01, µ = 0.02, Wilcoxon).

### HDAC5 rats had fewer reward-responsive neurons

**Figure 7.**
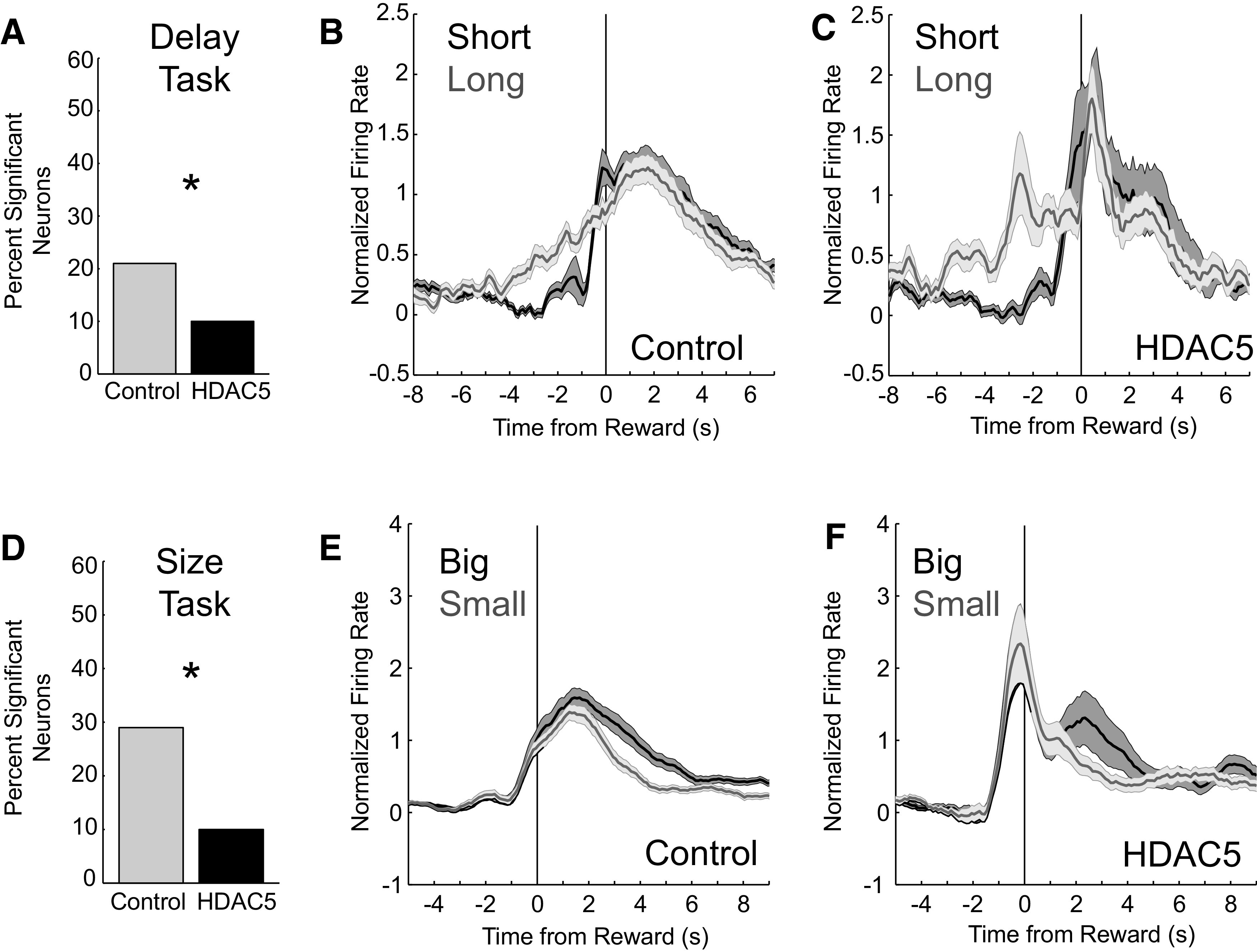
HDAC5 overexpression reduced counts of neurons that increased firing to reward delivery. Asterisks indicate significance. ***A***, Percentage of neurons that increased firing during the reward epoch (250 ms before reward delivery to 1 s after reward delivery). ***B***–***C***, Average firing of neurons that increased firing during the reward epoch in Control (*n* = 102, 21%) and HDAC5 rats (*n* = 54, 10%) in the delay task. Firing is aligned to reward delivery. ***D–F***, Same as ***A–C*** with data from the size task (Control = 104, 29%; HDAC5 = 49, 10%).

The above analyses suggest that HDAC5 overexpression elevated firing to stimuli before and during the initiation of the behavioral response, and firing during the sampling of stimuli was correlated with reaction time at the single-neuron level. During these analyses, we noticed that although firing was increased during presentation of odors and subsequent responding, firing during the delivery of reward appeared to be lower in HDAC5 rats than control rats (Fig. 5*A*,*E*). Based on this observation, we examined reward-related activity by asking how many neurons significantly increased firing during the anticipation and delivery of reward (reward epoch; *p* < 0.05; Wilcoxon). We found that 21% (*n* = 102; 37, 37, 16, 7, 5) cells in control rats increased firing to reward, whereas only 10% (*n* = 54; 17, 14, 13, 4, 4, 2,) increased responding after HDAC5 overexpression (Fig. 7*A*; Χ^2^ = 16.7, *p* = 4.4E-5, Χ^2^). However, there were no differences in baseline activity between these two populations of cells (*t*_(75_) = −0.963, *p* 0.338, unpaired *t* test).

The average firing of these neurons aligned to reward delivery is illustrated for both control and HDAC5 rats in Figure 7, *B* and *C*. As defined, firing increased over long delays until reward was delivered. Likewise, for size manipulations, we found 29% (*n* = 104; 49, 16, 14, 13, 12) of neurons from control rats increased during the reward epoch, whereas only 10% (*n* = 49; 19, 13, 7, 5, 5, 0) were observed after HDAC5 overexpression (Fig. 7*D*; Χ^2^ = 32.4, *p* = 1.26E-8, Χ^2^). Notably, consistent with the analysis during odor sampling described above, firing of HDAC5 overexpressed reward neurons also displayed prominent cue and movement-related firing before reward [Fig. 7*C*, HDAC5 (about −3 s); Fig. 7*F*, HDAC5 (about −1 s)].

## Discussion

Here, we examined the effects of DS HDAC5 overexpression on reward-guided decision-making and associated neural correlates in DLS. Our main findings are as follows. At the behavioral level, rats with HDAC5 overexpression showed decreased reaction time and inflexible behavior on both delay and size tasks; at the neural level, we observed an increase in the numbers of neurons that fired more to reward-predicting stimuli and cues that predicted short delayed reward but a decrease in the number of neurons that were responsive to anticipation and reward delivery following HDAC5 overexpression.

We observed a compelling increase and decrease in the selection of high-value rewards during the first and second block of trials in our HDAC5 rats. There are a number of potential explanations for this result. It is possible that learning in the first block of trials interfered with performance in the second block, or rats may have had trouble acquiring new associations once reward contingencies reversed in the second block. Although it is difficult to pinpoint the exact cause of our findings, we interpret these results to be evidence of behavioral inflexibility, based on previous studies examining the roles of HDACs and dorsal striatum in habit (Malvaez et al., 2018; Malvaez and Wassum, 2018). Decisions governed under habitual control are thought to be under the control of model-free systems that do not take into account task structures (e.g., frequent reversals), thus allowing animals to respond without deliberations and to develop associative behaviors more strongly and quickly. There is a trade-off, however, with behavioral flexibility. Here, the decrease in high-value reward selection may indicate such an inability to modify behavior once reward contingencies change.

Further support of this hypothesis may also come from the faster reaction times seen in our HDAC5 rats. In previous studies (Bryden et al., 2011; Burton et al., 2017, 2018; Brockett et al., 2018; Vázquez et al., 2019; Pribut et al., 2021) and our current study, we have used reaction time (i.e., how quickly rats leave the odor port after odor presentation) as a measure of how quickly rats decide which reward well to approach. Importantly, we observed no significant difference in movement times (i.e., how quickly rats enter the fluid well after odor port exit) in our HDAC5 rats, indicating our reaction time findings were not just a reflection of enhanced motor output. Together, our behavioral findings hint at a relationship between behavioral inflexibility and HDAC5 overexpression, although future studies will be needed to further explore other potential roles of HDAC5 in learning and memory mechanisms.

Most important, our findings suggest HDAC5 may be involved in abnormal decision-making behavior, a relationship that has thus far been largely unexplored. To date, only one other study has used a classic conditioning procedure to examine the role of HDAC3 in the formation of habitual behavior (Malvaez et al., 2018). This study, conducted by the Wassum lab, found that suppressing HDAC3 function, either through pharmacological or viral approaches in either DLS or DMS, facilitates habit formation, whereas potentiating HDAC3 function through viral-mediated HDAC3 overexpression in either dorsal striatal subregions prevents habit formation. Overall, these data indicate that in DS, HDAC3 negatively regulates habit formation. In contrast, we showed that HDAC5 overexpression in DS led to faster inflexible behavior, implicating a positive role of HDAC5 in regulating habitual behavior.

However, direct comparison between these two studies should be made with caution. Although all HDACs generally suppress gene expression, HDAC3 and HDAC5 belong to class I and class IIa HDAC, respectively, and they differ in many aspects that determine their distinct functions, such as structures, the protein complexes they form, downstream targets, cellular and tissue localization, enzymatic activities, and substrate specificities (De Ruijter et al., 2003; Seto and Yoshida, 2014). A question for future research is what cellular mechanisms—and differential effects on the regulation of downstream targets—lead to their distinct roles in habit and decision-making behavior.

Our single-unit recording data also provides the first evidence, to our knowledge, of a relationship between epigenetic mechanisms and the way in which neurons respond to different cues and reward, suggesting potential, dynamic connections among epigenetic events, neural activity, and changes in behaviors. With our current dataset, it is unclear whether HDAC5 overexpression produced changes in neural activity and subsequently behavior, or epigenetic changes produced behavioral changes that in turn altered patterns of neural activity within the DLS. However, we speculate that a finely orchestrated adaptation of gene expression across several classes of molecules (e.g., ion channels, glutamate receptors, transcription factors), as well as alterations in intracellular signaling pathways, would be required to modify the encoding properties of neurons. These relationships will be elucidated in future studies.

An intriguing application of our data is that HDAC5 and other epigenetic enzymes may serve as a critical link between psychiatric disorders and associated cognitive impairment. For example, we and others have previously studied how drugs of abuse disrupt normal decision-making and lead to impulsive, habitual behavior (Mendez et al., 2010; Burton et al., 2017, 2018; Vázquez et al., 2019; Pribut et al., 2021). Our lab has also shown that prior cocaine experience, even long after its acute effects have worn off, altered activity within DLS. These results are remarkably similar to the current study. A robust body of work has also examined HDACs in relation to drug addiction (Renthal and Nestler, 2009; Robison and Nestler, 2011; Rogge and Wood, 2013; Werner et al., 2021). In particular, we have previously shown that dysregulated HDAC5 in DS is relevant in an animal model of methamphetamine relapse (Li et al., 2018), leading to the question of whether the mechanisms between relapse and drug-induced impairments in decision-making are at all shared. Although addressing this question is beyond the scope of our current study, we are interested in future work where these issues are studied in tandem.

One limitation in the present study is that we overexpressed HDAC5 in both DMS and DLS; therefore, whether behavioral effects observed here require manipulations of the entire DS or specific subregions is unknown. However, based on our previous finding that decreased methamphetamine seeking is only observed after HDAC5 knockdown in both DMS and DLS, but not in either subregion alone (Li et al., 2018), we speculate that behavioral effects observed here require HDAC5 overexpression in the entire DS. Regarding neural correlates: Although we focused on examining DLS encoding here, we hope to include the DMS in future studies in light of emerging evidence implicating DMS in habitual control (Stalnaker et al., 2010; Malvaez and Wassum, 2018; Vandaele et al., 2019).

In sum, we demonstrated that rats with HDAC5 overexpression in DS demonstrated inflexible behaviors and altered associated neuronal encoding in DLS. Our findings contrast with examinations on the role of HDAC3 in DS in habit formation (Malvaez et al., 2018), indicating distinct mechanisms underlying decision-making and habitual control across different HDACs. Interestingly, our results were in line with previous observations of decision-making impairments after chronic cocaine use (Roesch et al., 2007; Burton et al., 2015, 2017, 2018; Brockett et al., 2018; Vázquez et al., 2019; Pribut et al., 2021). Such findings may suggest that HDACs could be a critical link between psychiatric disorders and associated cognitive impairment. Further studies will focus on the causal relationship among epigenetics, neural activity, and observed behavior, and how HDAC5 affects executive control and neuronal encoding in the context of drug addiction to further our understanding of the potential utility of HDACs in psychiatric treatments.
